# A Novel Single Domain Antibody Targeting FliC Flagellin of *Salmonella enterica* for Effective Inhibition of Host Cell Invasion

**DOI:** 10.3389/fmicb.2019.02665

**Published:** 2019-11-26

**Authors:** Jennifer Huen, Zhun Yan, Jeremy Iwashkiw, Shraddha Dubey, Maria C. Gimenez, Maria E. Ortiz, Saumil V. Patel, Michael D. Jones, Ali Riazi, Mauricio Terebiznik, Saeid Babaei, Dea Shahinas

**Affiliations:** ^1^AbCelex Technologies Inc., Mississauga, ON, Canada; ^2^Department of Biological Sciences, University of Toronto at Scarborough, Toronto, ON, Canada; ^3^Department of Cell and Systems Biology, University of Toronto at Scarborough, Toronto, ON, Canada

**Keywords:** single domain antibody, *Salmonella enterica*, pathogen, bacterial motility, bacterial invasion, binding affinity

## Abstract

The enteric pathogen, *Salmonella enterica* is a major cause of human gastroenteritis globally and with increasing bacterial resistance to antibiotics, alternative solutions are urgently needed. Single domain antibodies (sdAbs), the smallest antibody fragments that retain antigen binding specificity and affinity, are derived from variable heavy-chain only fragments (VHH) of camelid heavy-chain-only immunoglobulins. SdAbs typically contain a single disulfide bond simplifying recombinant protein production in microbial systems. These factors make sdAbs ideally suited for the development of effective anti-bacterial therapeutics. To this end, we generated an anti-Salmonella VHH library from which we screened for high affinity sdAbs. We present a novel sdAb (Abi-Se07) that targets the Salmonella virulence factor, FliC, required for bacterial motility and invasion of host cells. We demonstrate that Abi-Se07 bound FliC with a *K*_*D*_ of 16.2 ± 0.1 nM. In addition, Abi-Se07 exhibited cross-serovar binding to whole cells of *S. enterica* serovar Typhimurium, Heidelberg, and Hadar. Abi-Se07 significantly inhibited bacterial motility and significantly reduced *S. enterica* colonization in a more native environment of chicken jejunum epithelium. Taken together, we have identified a novel anti-Salmonella sdAb and discuss future efforts toward therapeutic development.

## Introduction

The Gram-negative bacterium, *Salmonella enterica* is a common contaminant in the food industry (Humphrey and Jørgensen, 2006; Crim et al., 2015). In chickens, *S. enterica* colonize the gastrointestinal tract, with little to no disease (Doyle and Erickson, 2006; Hugas and Beloeil, 2014; Mazengia et al., 2014; Wigley, 2014; Florez-Cuadrado et al., 2018). Consumption of bacteria-contaminated food products can cause non-typhoidal salmonellosis, an acute gastrointestinal illness in humans presenting with nausea, vomiting, diarrhea, and abdominal pain lasting 3–7 days (Crum Cianflone, 2009). Worldwide, it is estimated that the prevalence of salmonellosis is 3.4 million cases with over 680,000 deaths annually (Ao et al., 2015; Balasubramanian et al., 2018). With the large and growing market for broilers and the emergence of antibiotic resistant strains, alternative strategies to control *S. enterica* in livestock are needed in order to reduce the zoonotic infections.

The field of antibody therapeutics has expanded significantly in the past decade, with a record number of antibody drugs (12) approved in 2018 (Kaplon and Reichert, 2018). Technological advances have been integral in the identification and development of several formats of antibody based biologics, from full length antibodies that bind to tumor necrosis factor for the treatment of inflammatory diseases (Humira^®^, Salfeld et al., 1998; Alizadeh et al., 2015; Frenzel et al., 2016) to engineered fragments that neutralize neurotoxins produced by *Clostridia* (Miethe et al., 2014; Frenzel et al., 2016; Wang et al., 2016). Single domain antibodies (sdAbs) have become attractive molecules in therapeutic research. SdAbs are derived from the camelid heavy chain antibodies (HcAbs) where each antigen binding arm is composed of a variable heavy-chain-only domain (VHH) with three complementarity determining regions (CDRs) (reviewed in Arbabi-Ghahroudi, 2017). SdAbs are the smallest unit of the antibody (∼15 kDa) retaining binding specificity and affinity to antigen (De Meyer et al., 2014; Arbabi-Ghahroudi, 2017). The CDR3 of sdAbs is the most variable of all CDRs in both amino acid content and length (Muyldermans et al., 1994; Vu et al., 1997; Griffin et al., 2014). VHH CDR3 is typically longer than in conventional VHs and can access concave or cryptic epitopes not usually accessible by larger antibody fragments (De Genst et al., 2006; Schmitz et al., 2013). An additional, desired feature of sdAbs is that phage display and protein engineering are technically straightforward compared to conventional antibodies (Van Der Linden et al., 1999; Arbabi-Ghahroudi, 2017). SdAbs are ideal modular units for genetic construction of multi-valent or multi-specific formats (Els Conrath et al., 2001; De Bruin et al., 2017; Nosenko et al., 2017; Li et al., 2018). Production of sdAbs is also easier and more cost effective than full length IgGs since sdAbs generally contain a single disulfide bond and can be expressed to high yields in either the periplasm or cytosol of microbial systems (Zarschler et al., 2013; Arbabi-Ghahroudi, 2017; Shriver-Lake et al., 2017; Liu and Huang, 2018; Suzuki et al., 2018). These factors are important considerations in the development and production of antibody treatments.

We previously identified a sdAb (AbiBody^TM^) that binds to the flagella of *C. jejuni* and reduces bacterial colonization in chickens after sdAb treatment (Riazi et al., 2013). Here, we present the characterization of a novel sdAb, termed Abi-Se07, which targeted FliC, a flagellin protein of *S. enterica*. In an attempt to identify a sdAb that inhibits Salmonella invasion of host cells, FliC was chosen as a target. FliC is a virulence factor and the most abundant molecule of the flagella apparatus, forming the whip-like filament of the flagellum responsible for motility of Salmonella (Chevance and Hughes, 2008; Evans et al., 2014). The flagellar apparatus of Salmonella contributes to bacterial attachment to the host cell, facilitating the invasion process (Elhadad et al., 2015; Salehi et al., 2017 and reviewed in Chaban et al., 2015). We show Abi-Se07 bound to bacterial cells of *S. enterica* serovar Hadar, Heidelberg, Typhimurium, and directly to purified FliC of Typhimurium. We also show that Abi-Se07 impeded the motility of *S. enterica* serovar Hadar and Heidelberg. We demonstrate that treatment with Abi-Se07 reduced bacterial growth in a human and avian cell line. Importantly, *S. enterica* serovar Hadar growth was inhibited upon treatment with Abi-Se07 in chicken jejunum tissue sections. Overall, we present characterization of a novel anti-Salmonella sdAb.

## Materials and Methods

### Immunization, Library Generation, Panning, and Screening

*S. enterica* serovars Typhimurium, Hadar, Heidelberg, Enteritidis, Newport, Javiana, Senftenberg, Kentucky, Infantis, and Saint-Paul each at a concentration of 1 × 10^9^ CFU/mL in adjuvant (aluminum hydroxide, 2% Alhydrogel) were used for immunization of three male alpacas in a local farm (courtesy of Dr. Maurice Smith) by subcutaneous injections of 0.4 mL. Bacteria were cultured on Luria Broth (LB) agar plates, harvested in phosphate buffered saline (PBS, BioShop) and inactivated by heating at 65°C for 30′. Injections were done on days 1, 15, 22, 29, and 36. Total RNA was isolated from lymphocytes using RNAzol kit (Bioshop). Complementary DNA was synthesized with oligo (dT) primers using the SuperScript III First Strand cDNA synthesis kit (Invitrogen). Heavy chain variable regions were PCR amplified using forward (5′) primers MJ1, MJ2, or MJ3 and reverse primers (3′) CH2FORTA4 or CH2b3 (Arbabi-Ghahroudi et al., 2009; Baral et al., 2013). A second PCR reaction was conducted using the products of the first PCR reaction with the forward and reverse primers MJ7 and MJ8, respectively (Baral et al., 2013). These VHH PCR products were purified with Cycle Pure Kit (Omega Bio-tek), digested with *Sfi*I, and re-purified using the same kit. Twelve micrograms of digested DNA were ligated with 40 μg of Sfi-digested pADL-23c phagemid vector (Antibody Design Labs) using T4 DNA ligase (Promega), transformed into electrocompetent TG1 *Escherichia coli* cells (Lucigen Corporation), and a library with a size of 7.8 × 10^8^ transformants was obtained. Fifty clones were sequenced to analyze diversity and all fifty contained unique CDR sequences (Riazi et al., 2016). *E. coli* cells were infected with M13KO7 helper phage (New England Biolabs) in the presence of ampicillin (100 ng/mL) and kanamycin (25 ng/mL) and the phage library was precipitated in final concentration of 4% polyethylene glycol 8000 in 0.5 M NaCl and resuspended in PBS. Panning was performed on 5 μg/mL of FliC (Sigma-Aldrich) coated on a 96-well immunosorbent plate and blocked with 0.5% bovine serum albumin (BSA) for 2 hrs. Approximately 2 × 10^12^ phage particles were added to BSA-only-coated wells for 1 h at 37°C to select out non-specific binders. The supernatant was then transferred to FliC-coated wells and the plate was incubated for 2 h at 37°C. Wells were washed 5× with PBST (PBS, 0.1% Tween-20) and bound phages eluted with 0.1 M triethylamine and neutralized with 1M Tris-HCl, pH 7.4. Eluted phages were amplified by infecting exponentially growing TG1 *E. coli* for 30′ at 37°C and then with helper phage for an additional 30′. Phages were precipitated after 16 h incubation and used for the next round of panning. Three additional panning rounds were conducted following the same conditions except washing was increased 7, 10 and, 12 times with phosphate-buffered saline with 0.1% Tween-20 (PBST) for the second, third and fourth rounds, respectively. After the fourth round, phage-infected TG1 cells were plated, 96 randomly picked colonies were grown for enzyme linked immunosorbent assays (ELISAs) against FliC.

For clonal screening by ELISAs, 2.5 μg/mL of FliC was coated on a 96-well immunosorbent plate and blocked with BSA. The 96 phage clones were then applied and the plate was incubated for 1 h at room temperature (RT). After washing with PBST, horse radish peroxidase (HRP)-conjugated anti-M13 monoclonal antibody (GE Healthcare) at 1:5000 v/v concentration was applied and the plate was incubated for 30′ at RT, shaking. Wells were washed with PBST and 3, 3′, 5, 5′-Tetramethylbenzidine (TMB) peroxidase substrate (Thermo Fisher) was added for colorimetric quantification. Reactions were stopped with 1M hydrochloric acid and absorbance was read at 450 nm using a Cytation 5 plate reader (Biotek).

### Cloning, Protein Expression and Purification

The Abi-Se07 gene was synthesized (Integrated DNA Technologies) and cloned into the pET27b bacterial expression vector by Gibson assembly (Gibson et al., 2009). The sdAb construct was expressed periplasmically in BL21 (DE3) *E. coli* with a C-terminal c-myc tag followed by a His_6_-tag. Cells were grown to mid-log phase in Terrific Broth (TB) media and induced with 1 mM isopropylthio-β-galactoside (IPTG), extracted by osmotic shock and purified by immobilized Nickel affinity chromatography. Peak elutions were dialyzed into PBS and quantified by UV spectrophotometry by absorbance at 280 nm.

### Bacterial Binding by ELISAs

Heat-inactivated *S. enterica* serovar Hadar, Heidelberg Typhimurium WT or *flhD* mutant EG11308 (Table 1) were coated onto immunosorbant plates at 4°C for 16 hrs. Plates were blocked with 5% skim milk for 2 h at RT. SdAb constructs were then applied at 250, 25, 2.5, 0.25, 0.025, and 0.0025 nM for *S. enterica* and plates were incubated for 1 hr at RT. Plates were then washed 6× with PBST (0.1% Tween-20) and HRP-conjugated anti-His polyclonal antibody (Bethyl laboratories) was applied at 1:5000 v/v concentration for 30′ at RT. Plates were washed again 6× with PBST and TMB peroxidase substrate was added for colorimetry quantification. Absorbance was read at 450 nm using Cytation 5.

**TABLE 1:** Strains used in this study.

**Strain**	**Genotype or source**	**References**
*S. enterica* serovar Typhimurium 14028s WT	American type culture collection	Park et al., 2015
*S. enterica* serovar Typhimurium EG11308 flhD	flhD:Tn10dCm in 14028s background	Park et al., 2015
*S. enterica* serovar Hadar	Salmonella genetic stock centre	Riazi et al., 2016
*S. enterica* serovar Heidelberg	Salmonella genetic stock centre	Riazi et al., 2016

### Salmonella Motility Assays

Exponentially growing *S. enterica* serovar Hadar or Heidelberg were diluted to 0.01 OD_600_ and incubated with Abi-Se07 or anti-Campylobacter irrelevant sdAb (for Heidelberg experiment) for 10′ at RT. One microliter of the mixture was spotted on Muller Hinton-soft agar (1.5% MH media, 0.5% glucose, 0.5% agar) in 100 mm-diameter petri dishes and let dry for 10′. Plates were incubated for 16–25 h at 37°C in 5% CO_2_ (Forma Direct heat incubator, Thermo Scientific) and imaged. Area of cell spread was quantified using ImageJ software and analyzed using multiple comparison ANOVA (Prism, GraphPad).

For dose-dependent motility assays, *S. enterica* was treated with Abi-Se07 at varying concentrations for 10′ at RT and the mixture was spotted on the corner of MH-soft agar pad at 0.3% agar in a 96-well plate. After incubation for 16 hrs at 37°C, each well was applied with AlamarBlue (530–560 ex/590 em, Thermo Fisher), which emits a red-fluorescent signal when it becomes reduced by metabolically active cells. The plate was incubated for 30′ at 37°C, and fluorescence was read at the center of each agar pad using Cytation 5 plate reader. Fully motile cells would spread across the agar pad and exhibit higher fluorescence signal at the center of the pad while non-motile cells would remain at the corner of the pad and exhibit lower signal (Rasmussen et al., 2011). Relative fluorescence units (RFU) were plotted as a function of protein concentration and statistical significance was analyzed by multiple comparison ANOVA (Prism, GraphPad).

### Assaying Salmonella Adhesion/Invasion of Host Cells

GFP-expressing *S. enterica* serovar Hadar or non-fluorescent *S. enterica* serovar Hadar were treated with 500 μg/mL of Abi-Se07 and incubated for 30′ at 37°C. Then, confluent HeLa cells (ATCC^®^ CCL-2^TM^) or QM7 cells (ATCC^®^ CRL-1962^TM^) were infected at a multiplicity of infection (MOI) of 50 with GFP expressing *S. enterica* serovar Hadar, or *S. enterica* serovar Hadar, respectively. To quantify cell-associated Salmonella in HeLa cells, fluorescence images were taken with Cytation 5 and fluorescent bacteria quantified. To measure intracellular bacteria, HeLa or QM7 cells were then treated with 100 μg/mL gentamicin and incubated for 1 h at 37°C. Gentamicin is a non-membrane permeable antibiotic and will kill non-internalized bacteria. Finally, cells were washed 3× with PBS. For HeLa cells, fluorescence images were taken with Cytation 5 and fluorescent bacteria quantified. For QM7 cells, 0.1% saponin solution was added and allowed to incubate with cells for 10′ at 37°C. The mixture was then vortexed and homogenized for cell lysis. Lysates were serially diluted and plated on LB agar for CFU counting. Statistical analysis was performed using unpaired student *t*-test.

### Assaying Salmonella Invasion of Chicken Intestinal Epithelium

For infection of chicken jejunum, intestinal sections (Lloyd Weber Veterinary Clinic, Guelph, Ontario) were treated with 3× Hank’s Balanced Salt Solution (HBSS) and gentamicin at 50 μg/mL final concentration. Sections were cut with 4 mm diameter biopsy punches and placed into 24-well plate with 1 mL of Dulbecco’s Modified Eagle Medium (DMEM), 10% fetal bovine serum (FBS), and gentamicin. The plate was incubated at 37°C for 1 h, then washed with HBSS at least 3×. *S. enterica* at OD_600_ of 0.5 was treated with 500 μg/mL of Abi-Se07, incubated at 37°C for 20′, applied to the jejunum tissues, and the plate was incubated for another hour. Tissues were then washed 2× with HBSS and gentamicin was applied again for 15′. Tissues were washed 3× with HBSS and incubated for 30′ in DMEM and 10% FBS. Total DNA was extracted from the samples for qPCR of the Salmonella ttr locus using the method described by Malorny et al. (2004).

## Results

### Identification of a Novel Anti-Salmonella sdAb: Abi-Se07

Three male alpacas were subcutaneously immunized with 10 serovars of heat-inactivated cells of *S. enterica* to select for antibodies with inter-serovar cross-reactivity. A phage-displayed VHH library was generated from lymphocyte total RNA. We performed panning/enrichment of the phage library against FliC of serovar Typhimurium using ELISA. ELISAs and Sanger sequencing were performed on 96 phage clones randomly chosen after the last round of panning. We identified 18 unique sequences, grouped based on CDR sequences that were specific binders by ELISA (Table 2).

**TABLE 2:** Consensus amino acid sequences of CDR1, CDR2, and CDR3 of the five groups of 18 anti-Salmonella VHHs.

**CDR1**		
Group 1	GRX_1_FSX_2_KP	0A08; 1B08; 0A07; 1E05; 0H12; 1H07; 1E03; 0F09; 4D01; 0D12
Group 2	GLDFSSYA	4F10; 1E08; 3B04; 4F12
Group 3	GIIFSINA	2A09; 1G06
Group 4	GRSFSLYG	0A09
Group 5	GSIFSGDA	4C10
X_1_ = T or S; X_2_ = V or K		
**CDR2**		
Group 1	ASX_3_TGVST	0A08; 1B08; 0A07; 1E05; 0H12; 1H07; 1E03; 0F09; 4D01; 0D12
Group 2	ISRFGGRL	4F10; 1E08; 3B04; 4F12
Group 3	ISAYDHT	2A09; 1G06
Group 4	ISGSGLATS	0A09
Group 5	IGKEGDT	4C10
X_3_ = F or Y		

**CDR3**		
Group 1	AGTX4RTLWGSKWRDX5X6EYEY	0A08; 1B08; 0A07; 1E05; 0H12; 1H07; 1E03; 0F09; 4D01; 0D12
Group 2	AADRRSGLGTSKEYDY	4F10; 1E08; 3B04; 4F12
Group 3	NVDEIRKF	2A09; 1G06
Group 4	AQRWTSGTIARATGEYGY	0A09
Group 5	ATFEERPQPSYVY	4C10
X_4_ = T or L; X_5_ = V or R; X_6_ = L or R		

**FIGURE 1:**
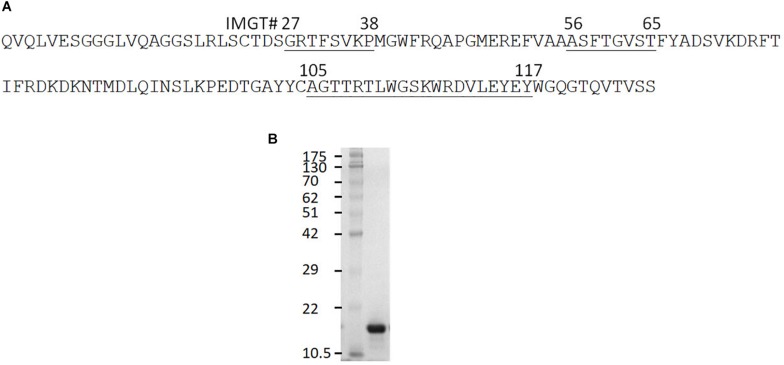
Anti-Salmonella Abi-Se07 sequence and purification. **(A)** Amino acid sequence of Abi-Se07 with CDR1-3 labeled according to IMGT numbering. **(B)** Denaturing SDS-PAGE gel of Abi-Se07 after purification by IMAC.

The clone 0A07 was selected based on neutralization of most Salmonella strains tested. From here, it will be referred to as Abi-Se07. It belongs to group 1 and contains a CDR3 20 amino acids in length (Figure 1A), longer than the average CDR3 of sdAbs at 13–18 amino acids in length (Muyldermans et al., 1994; Vu et al., 1997; Griffin et al., 2014). As a visualization exercise of Abi-Se07 binding to FliC, we modeled Abi-Se07, performed docking simulations, and *in silico* alanine scanning (Supplementary Figure S1 and Supplementary Table S1, respectively). The CDR loops of the model are likely conformationally variable, consistent with the lowered model quality around CDR regions, lowest around CDR3 (Supplementary Figure S1C), indicating the inaccuracy of the model. These *in silico* exercises merely provide a to-scale visualization.

### Abi-Se07 Binds to Cells of *S. enterica* Serovar Heidelberg, Typhimurium, and Hadar

**FIGURE 2:**
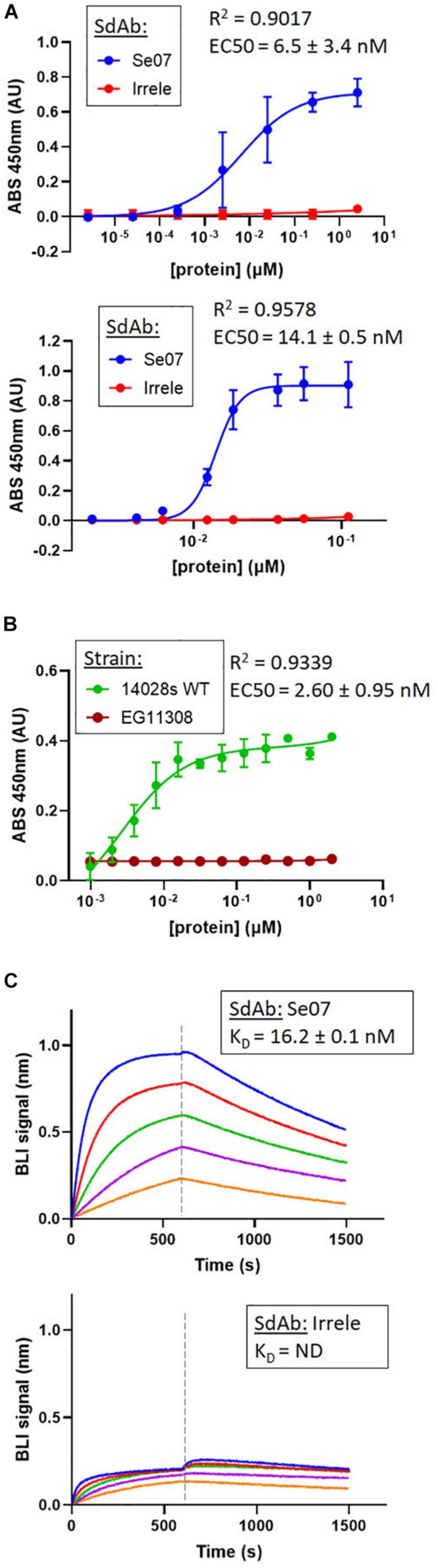
Abi-Se07 binds to *S. enterica* serovar Hadar, serovar Typhimurium, and FliC protein. **(A)** Abi-Se07 (blue) and non-target anti-Campylobacter irrelevant sdAb (red) were assayed for binding to whole cells of *S. enterica* serovar Hadar (top panel) and Heidelberg (bottom panel) by ELISA. Error bars represent standard deviation of three replicates. **(B)** Abi-Se07 was assayed for binding to *S. enterica* serovar Typhimurium (blue) and to the EG11308 *fliD* mutant of Typhimurium (red). EC50 values were determined using non-linear regression one site binding model and indicated with the standard error of the fit. Error bars represent standard deviation of two independent replicates. **(C)** Sensorgrams of BLI experiments measuring the binding of Abi-Se07 (top panel) and irrelevant sdAb (bottom panel) to FliC flagellin of *S. enterica* serovar Typhimurium. SdAb was immobilized to nickel-charged biosensors and dipped into different concentrations of FliC: 250, 125, 62.5, 31.3, and 15.6 nM (blue to orange). Dashed line separates FliC association and FliC dissociation. *K*_D_ value is indicated with standard error of the fit.

Based on the docking results, we tested whether Abi-Se07 exhibited cross-serovar binding on three serovars, *S. enterica* serovar Heidelberg, Typhimurium, and Hadar, of which the latter two are globally prevalent in outbreaks (Lan et al., 2009; Bogomolnaya et al., 2014). We expressed and purified Abi-Se07 (Figure 1B) and conducted ELISAs on whole cells of *S. enterica* serovars Hadar, Heidelberg, and Typhimurium (Figures 2A,B). Abi-Se07 exhibited binding to serovar Hadar and Heidelberg while no binding was detected for the off-target control sdAb, Irrelevant, which was developed to bind flagella of *C. jejuni* (Hussack et al., 2014). Abi-Se07 also exhibited binding to serovar Typhimurium and not to the *flhD* mutant (Park et al., 2015). We determined, using biolayer interferometry (BLI), that Abi-Se07 binds to purified FliC, with a *K*_*D*_ of 16.2 ± 0.1 nM (Figure 2C, upper panel), while the irrelevant sdAb did not (lower panel). These results indicate that Abi-Se07 exhibits cross-serovar binding, consistent with the docking simulations.

### Bacterial Motility Is Inhibited With Abi-Se07 Treatment

**FIGURE 3:**
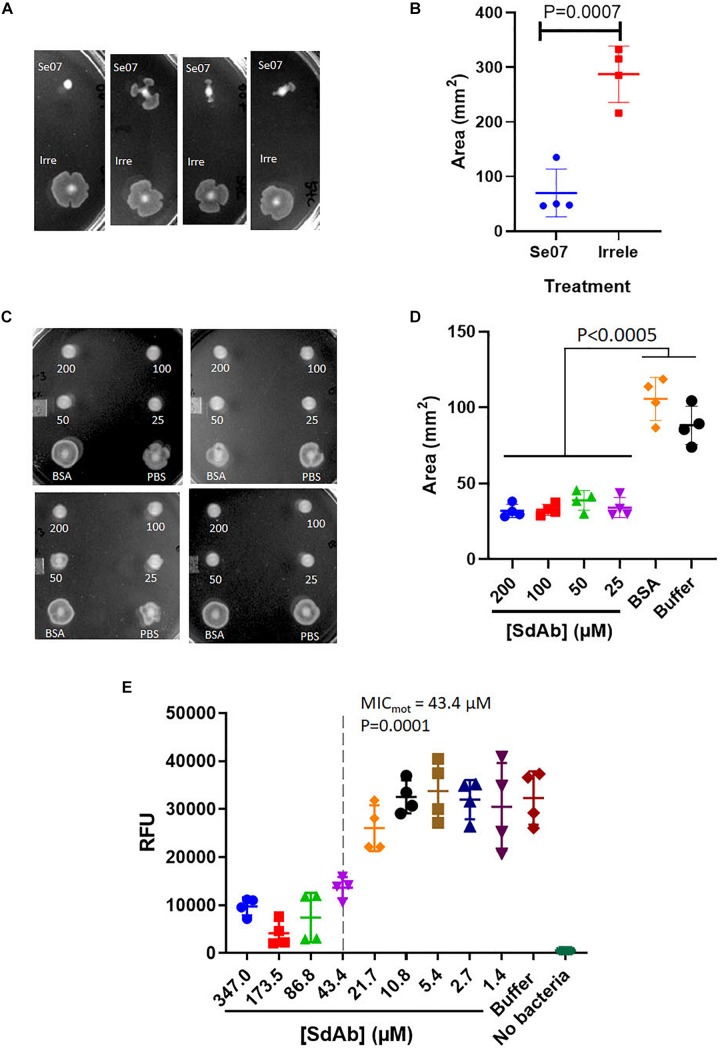
Abi-Se07 inhibits the motility of *S. enterica* serovar Heidelberg and Hadar. **(A)** *S.* Heidelberg and **(C)** *S.* Hadar on soft agar plate. Four replicates are shown. **(B)** Quantification of area of *S. enterica* serovar Heidelberg treated with Abi-Se07 or non-target anti_Campylobacter irrelevant sdAb. **(D)** Quantification of area of *S.* Hadar treated with Abi-Se07, 75 μM BSA, or PBS buffer. Statistical significance between Abi-Se07 treatment and BSA or Buffer control is indicated by *p* < 0.0005 using multiple comparisons ANOVA. Error bars represent standard deviation of the mean of four independent replicates. **(E)** Dose-dependent response of Se07 on motility of *S.* Hadar. Minimum inhibitory concentration of motility (MIC_mot_) is shown as dotted line with *p* = 0.0001 compared to Buffer treatment control. Error bars represent standard deviation of four independent replicates.

We next assessed Abi-Se07-dependent reduction of *S. enterica* motility by preincubating bacteria with Abi-Se07 and plating the mixture on soft agar (Figure 3). *S. enterica* serovar Typhimurium was difficult to assay, exhibiting high degree of variability in motility (Supplementary Figure S2). For this reason, we focused on serovar Heidelberg and Hadar for our motility studies. Treatment of these strains with Abi-Se07 resulted in significant motility inhibition compared to non-target anti-Campylobacter irrelevant sdAb (Figures 3A,B), BSA treatment or buffer control (Figures 3C,D), suggesting that Abi-Se07 affects bacterial motility by specifically binding FliC. To quantify the magnitude of motility inhibition, dose-dependent motility assays were conducted on *S.* Hadar (Figure 3E). Minimum inhibitory concentration of motility (MIC_mot_) was determined as the lowest concentration of sdAb (43.4 μM) that yielded significant reduction in motility.

### Bacterial Invasion of Eukaryotic Cells Is Reduced After Treatment With Abi-Se07

**FIGURE 4:**
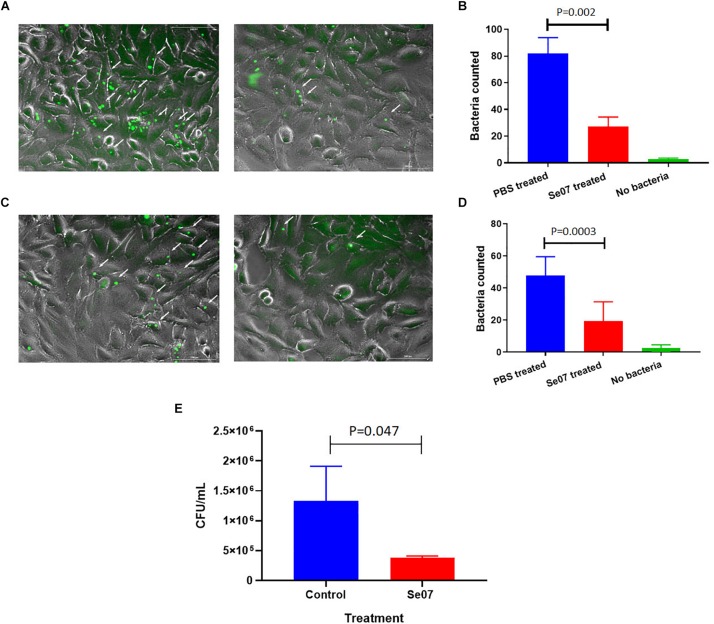
Salmonella invasion of human and avian cell lines is reduced with Abi-Se07 treatment. **(A)** GFP-expressing *S. enterica* serovar Hadar treated with buffer (left panel) or 500 μg/mL Abi-Se07 (right panel) were used to infect HeLa cells and imaged by fluorescence microscopy, pre-gentamicin. Fluorescent bacteria are shown beside the white arrows. **(B)** Bar graph of fluorescent bacteria counts quantified from 7 different images. PBS treatment is in blue, Abi-Se07 treatment in red, and no bacteria in green. **(C)** *S. enterica* serovar Hadar*-*infected HeLa cells were treated with gentamicin to remove extracellular bacteria to assess the number of intracellular bacteria. Fluorescence microscopy images are shown for buffer treatment (left panel) and Abi-Se07 treatment (right panel). **(D)** Bar graph of fluorescent bacteria counts, post-gentamicin, quantified from seven different images. PBS treatment is in blue, Abi-Se07 treatment in red, and no bacteria in green. Error bars represent the standard deviation. Statistical significance was determined by unpaired student *t*-test. **(E)** Bar graph of mean CFU counts of intracellular *S. enterica* in the presence and absence of Abi-Se07 treatment at 500 μg/mL. QM7 cells were infected with Abi-Se07-treated *S. enterica* (red) or buffer-treated *S. enterica* (blue) and intracellular bacteria were quantified 1 h p.i. after gentamicin treatment. Error bars represent standard deviation of three independent replicates.

*S. enterica* motility is essential for attachment and invasion of eukaryotic cells (Elhadad et al., 2015; Salehi et al., 2017). To evaluate the effect of Abi-Se07 on *S. enterica* infectivity, attachment and invasion assays were performed using human and avian cell lines. We co-incubated HeLa cells with GFP-expressing *S. enterica* serovar Hadar treated with Abi-Se07 and the number of adhered or intracellular bacteria were quantified by fluorescence microscopy (Figures 4A,B). A significant reduction of cell-associated bacteria were observed in Abi-Se07-treated bacteria versus PBS-treated in both pre-gentamicin (Figures 4A,B) and post-gentamicin experiments (Figures 4C,D). Abi-Se07 treatment resulted in a 3-fold reduction of bacteria in the pre-gentamicin experiment and a 2.5-fold reduction of internalized bacteria in the post-gentamicin experiment. Next, the effect of Abi-Se07 on *S. enterica* infection was assessed in the avian cell line, QM7, by quantifying intracellular bacteria 1-h post-infection (1 h p.i.). Treatment of *S. enterica* with Abi-Se07 resulted in 3.5-fold reduction of internalized bacteria compared to control (Figure 4E). These results indicate that Abi-Se07 treatment of *S. enterica* inhibits invasion of eukaryotic cells.

### Abi-Se07 Dependent Reduction of *S. enterica* Invasion of Chicken Gut Tissue

**FIGURE 5:**
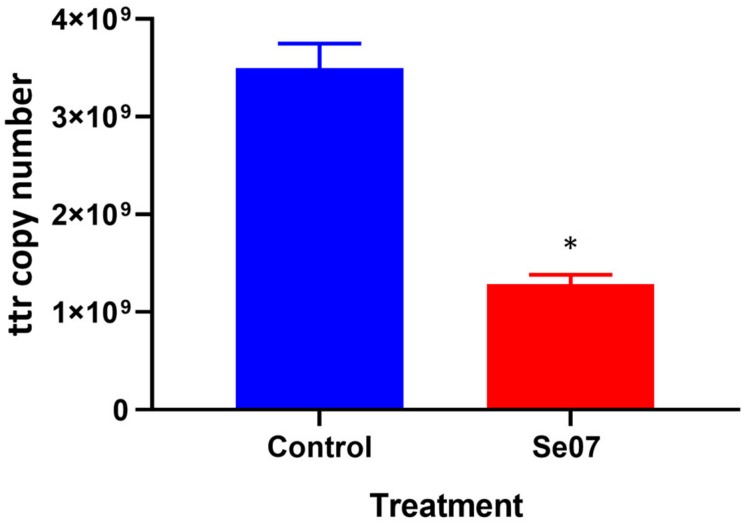
Salmonella invasion of chicken jejunum tissue is reduced with Abi-Se07 treatment. Chicken jejunum sections were infected with Abi-Se07-treated bacteria (at 500 μg/mL) and analyzed by qPCR of the ttr genetic locus. Bar graph shows buffer treatment in blue and Abi-Se07 treatment in red. Error bars represent standard deviation of two replicates. Statistical significance of ^∗^*p* = 0.0074 was calculated using unpaired student’s *t*-test.

To investigate the effect of Abi-Se07 using a relevant biological sample, we performed invasion assays *ex vivo* with chicken intestinal epithelium. Chicken jejunum sections were co-incubated with Abi-Se07-treated *S. enterica* serovar Hadar. To reduce non-specific signal from other bacteria present in the tissue sections, the tetrathionate reductase (ttr) genetic locus specific to Salmonella (Malorny et al., 2004) was used as a reporter for quantitative PCR. The control treatment exhibited 3.5 × 10^9^ ± 0.3 × 10^9^ ttr DNA copies while the Abi-Se07 treatment showed 1.3 × 10^9^ ± 0.1 × 10^9^ DNA copies, a 2.7-fold reduction (Figure 5). Taken together, Abi-Se07 has an inhibitory effect on *S. enterica* invasion in natural host tissue.

## Discussion

An anti-Salmonella library of single domain antibodies was created by immunizing alpacas with 10 different serovars of *S. enterica*. FliC of serovar Typhimurium was chosen as this serovar is one of the most commonly found serovars in both humans and livestock (Lan et al., 2009; Bogomolnaya et al., 2014). FliC was chosen as a target because it is an important and abundant virulence factor of *S. enterica* and immunogenic in humans (Alaniz et al., 2006).

We demonstrated that by panning with FliC, we could extract a sdAb, Abi-Se07, with a *K*_D_ of 16.2 nM, within the mid-nM to high-pM range for sdAbs as reported by others (Muyldermans and Lauwereys, 1999; Demeestere et al., 2016; Sulea et al., 2018). Abi-Se07 contains a longer than average CDR3 loop (Figure 1), and could suggest that its CDR3 forms a convex paratope to access a cryptic epitope on FliC as observed with other long CDR3 sdAbs (Stijlemans et al., 2004; De Genst et al., 2006; Schmitz et al., 2013). The accessibility of a cryptic epitope suggests that Abi-Se07 may target a conserved region on FliC, supported by the fact that Abi-Se07 exhibited cross-serovar binding to whole cells of *S. enterica* serovar Hadar, Heidelberg, and Typhimurium with similar EC50 values. Binding of multiple antigen isoforms is a desired property in therapeutic antibodies since the sdAb can remain effective against highly mutagenic antigens. For example, the sdAb was developed targeting the variant-specific surface glycoprotein (VSG) of the parasite *Trypanosome* where a number of different VSGs were effectively bound by the sdAb (Stijlemans et al., 2004). Our docking simulations with Abi-Se07 model identified a conserved region on FliC that may be accessible by the sdAb with favorable binding energy (Supplementary Figure S1B). However, these simulations remain to be validated by empirical methods.

We demonstrated Abi-Se07 treatment reduced *S. enterica* motility and invasion of different cell types. Motility assays conducted with *S. enterica* serovars Hadar and Heidelberg were more consistent than with Typhimurium and could indicate differences in environmental sensing, motility regulation, and other virulence-related functions between the serovars (Suez et al., 2013; Dhanani et al., 2015). Using *S. enterica* serovar Hadar, we showed that bacterial invasion is significantly reduced with Abi-Se07 treatment, which may still be due to motility inhibition. This was observed on non-native host cells (HeLa and QM7 cell lines) and native host tissue (jejunum epithelium of chickens).

With KD in nanomolar range, it was unexpected that MIC would be in micromolar range based on the proposed mechanism of action and the Scatchard equation. We posit that quantification error is minimal as two detection methods gave consistent protein concentrations and aggregation of sdAb in the assay conditions is unlikely as Tm ∼60°C and antibodies are relatively stable proteins. It is possible that this observation is due to bacterial protease-associated inactivation of sdAbs. Another alternative is that Abi-Se07 is binding flagella, disrupting motility and resulting in reduced host cell invasion. Therefore, the functional concentration of Abi-Se07 may be low due to the abundance of the flagellar units and the small size of the antibody.

A statistically significant reduction was observed upon Abi-Se07 treatment on bacterial invasion in host cells, which may also be due to inhibition of motility on mucus as a result of Abi-Se07 binding the flagella. Efforts are underway to further develop this antibody into a therapeutic biologic and the results demonstrated here pave the way for future sdAb development such as affinity maturation by directed evolution, structure-guided engineering, and synthetic library designs.

## Animal Tissues

The broiler chicken intestinal tissue sections for our *ex vivo* studies were obtained from chickens that had been already euthanized in a private practice: the Guelph Poultry Veterinary Services under the guidance of Dr. Lloyd Weber, DVM, who is affiliated with the Poultry Health Research Network of the University of Guelph, and operates under the guidelines of the University of Guelph Animal Care Committee.

## Data Availability Statement

All datasets generated for this study are included in the article/Supplementary Material.

## Author Contributions

JH, ZY, JI, SD, MG, MO, AR, MT, SB, and DS designed the experiments. JH, ZY, JI, SD, MG, SP, MJ, MO, AR, and DS performed the experiments and analyzed the data. JH, JI, MJ, SD, MG, MO, MT, SB, and DS wrote, reviewed, and modified the manuscript.

## Conflict of Interest

ZY, JI, SD, SP, MJ, AR, SB, and DS were employees of AbCelex Technologies Inc. AR, DS, SB, and ZY are inventors on an issued patent regarding the anti-Salmonella phage-display library and the corresponding antibodies. SB is a shareholder of AbCelex Technologies Inc. The majority of the funding for this research was provided by AbCelex Technologies Inc. and the research was conducted in their laboratories.

The remaining authors declare that the research was conducted in the absence of any commercial or financial relationships that could be construed as a potential conflict of interest.

## Supplementary Material

FIGURE S1 **(A)** Alignment of *S. enterica* serovar Hadar (RAT91061.1), Typhimurium (AHA06007.1), Heidelberg (AAT81640.1), Enteritidis (ERH38463.1), Newport (APH26739.1), Javiana (OLP51747.1), Senftenberg (ABR18492.1). Kentucky (KWV07712.1), Infantis (OSY33027.1), and Saint-Paul (KNU33033.1). Domains D0, D1, D2, and D3 are colored black, gray, cyan, and green, respectively. Underlined region corresponds to residues within 4 Å of Abi-Se07 CDRs in the simulated docking solution. **(B)** Visualization of docking of Abi-Se07 to FliC. This docking is inaccurate due to low quality modeling of CDRs (see **C**) and is provided merely as a to-scale visualization. **(C)** SWISS-MODEL QMEAN quality score relative to all non-redundant structures in the protein data bank (left and right panels). Local quality estimate along the length of the sdAb polypeptide (middle panel). Quality estimate declines around residue 100 to 120, corresponding to CDR3.

FIGURE S2 Motility of *S. enterica* was assessed for serovars Typhimurium and Heidelberg. Three replicates were conducted for each set of experiments. In the presence of BSA, PBS or irrelevant sdAb, Typhimurium exhibited inconsistent motilities while Heidelberg exhibited consistent and reproducible motilities with the control treatment.

TABLE S1 Computational alanine scanning run with Robetta (www.bakerlab.org).
