# Supplementary material for: A Novel Single Domain Antibody Targeting FliC Flagellin of Salmonella enterica for Effective Inhibition of Host Cell Invasion
Source: Front Microbiol. 2019 Nov 26;10:2665. doi: 10.3389/fmicb.2019.02665 (PMC6901939; doi:10.3389/fmicb.2019.02665)
Supplement: FIGURE S1 — (A) Alignment of S. enterica serovar Hadar (RAT91061.1), Typhimurium (AHA06007.1), Heidelberg (AAT81640.1), Enteritidis (ERH38463.1), Newport (APH26739.1), Javiana (OLP51747.1), Senftenberg (ABR18492.1). Kentucky (KWV07712.1), Infantis (OSY33027.1), and Saint-Paul (KNU33033.1). Domains D0, D1, D2, and D3 are colored black, gray, cyan, and green, respectively. Underlined region corresponds to residues within 4 Å of Abi-Se07 CDRs in the simulated docking solution. (B) Visualization of docking of Abi-Se07 to FliC. This docking is inaccurate due to low quality modeling of CDRs (see C) and is provided merely as a to-scale visualization. (C) SWISS-MODEL QMEAN quality score relative to all non-redundant structures in the protein data bank (left and right panels). Local quality estimate along the length of the sdAb polypeptide (middle panel). Quality estimate declines around residue 100 to 120, corresponding to CDR3. [file Data_Sheet_1.docx]

**Supplementary Material**

**Supplementary Methods**

**Antigen binding by biolayer interferometry**

SdAb constructs at 400 nM were immobilized to a nickel-coated BLI biosensor (ForteBio) and dipped into either heat inactivated *S. enterica* serovar Hadar, FliC of *S. enterica* (Sigma-Aldrich), or flagella of *C. jejuni* (purified according to (Newell, McBride, and Pearson 1984)). After loading for 10’, the biosensors were base lined in 1 x Kinetics PBS buffer (ForteBio) for 5’ then dipped into antigen solution. For K_D_ determination, FliC was assayed at the following concentrations: 500, 250, 125, 62.5, 31.3, and 15.7 nM and flagella assayed at: 2400, 1200, 600, 300, 150, and 75 nM. For assessing bacteria binding, BSA was used in the mobile phase as a negative control. BLI signal (nm) was recorded with the Octet RED96 BLI (ForteBio).

**Thermostability assays by differential scanning fluorimetry**

Differential scanning fluorimetry was performed as previously described (Huynh and Partch 2015). Briefly, Sypro orange (5000 x, ThermoFisher) was added to proteins at a concentration of 6 x and scanned using 7500 Fast Dx RT-PCR instrument (Applied Biosystems). ROX (6-carboxy-X-rhodamine) was chosen as the fluorescence reporter and none for quencher. Method was set for 1 ⁰C increase per minute from 25 ⁰C to 95 ⁰C. Proteins at 0.9 mg/mL were scanned with five replicates for each construct.

**Molecular docking**

The structure of Abi-Se07 was modeled using SWISS-MODEL. Domains D2-D3 of FliC (Yonekura, Maki-Yonekura, and Namba 2003) was used for docking of Abi-Se07 using SnugDock (Sircar and Gray 2010). Docking was conducted with 100 iterations and the lowest energy solutions (Supplementary Table1) were analyzed and visualized as part of the filament molecule.

The homology model was generated by Swiss-Model with quality score in good range relative to all structures in Protein Data Bank. The quality score is an estimate of C-beta angle energies, all atom energies, solvation, and torsion compared to experimentally determined protein crystal structures. Quality of the model is higher in the framework than the CDR regions, which is not surprising as these regions are expected to be flexible and structurally different than CDRs of structurally available antibodies.

**A**

ERH38463.1 MAQVINTNSLSLLTQNNLNKSQSSLSSAIERLSSGLRINSAKDDAAGQAIANRFTSNIKG 60

AAT81621.1 MAQVINTNSLSLLTQNNLNKSQSSLSSAIERLSSGLRINSAKDDAAGQAIANRFTSNIKG 60

OLP51747.1 MAQVINTNSLSLLTQNNLNKSQSALGTAIERLSSGLRINSAKDDAAGQAIANRFTANIKG 60

APH26739.1 MAQVINTNSLSLLTQNNLNKSQSALGTAIERLSSGLRINSAKDDAAGQAIANRFTANIKG 60

KNU33033.1 MAQVINTNSLSLLTQNNLNKSQSALGTAIERLSSGLRINSAKDDAAGQAIANRFTANIKG 60

RAT91061.1 MAQVINTNSLSLLTQNNLNKSQSALGTAIERLSSGLRINSAKDDAAGQAIANRFTANIKG 60

OSY33027.1 MAQVINTNSLSLLTQNNLNKSQSALGTAIERLSSGLRINSAKDDAAGQAIANRFTANIKG 60

KWV07712.1 MAQVINTNSLSLLTQNNLNKSQSALGTAIERLSSGLRINSAKDDAAGQAIANRFTANIKG 60

AHA06007.1 MAQVINTNSLSLLTQNNLNKSQSALGTAIERLSSGLRINSAKDDAAGQAIANRFTANIKG 60

AAT81640.1 MAQVINTNSLSLLTQNNLNKSQSALGTAIERLSSGLRINSAKDDAAGQAIANRFTANIKG 60

***********************:*.:****************************:****

ERH38463.1 LTQASRNANDGISIAQTTEGALNEINNNLQRVRELSVQATNGTNSDSDLKSIQDEIQQRL 120

AAT81621.1 LTQASRNANDGISIAQTTEGALNEINNNLQRVRELSVQATNGTNSDSDLKSIQDEIQQRL 120

OLP51747.1 LTQASRNANDGISIAQTTEGALNEINNNLQRVRELAVQSANSTNSQSDLDSIQAEITQRL 120

APH26739.1 LTQASRNANDGISIAQTTEGALNEINNNLQRVRELAVQSANSTNSQSDLDSIQAEITQRL 120

KNU33033.1 LTQASRNANDGISIAQTTEGALNEINNNLQRVRELAVQSANSTNSQSDLDSIQAEITQRL 120

RAT91061.1 LTQASRNANDGISIAQTTEGALNEINNNLQRVRELAVQSANSTNSQSDLDSIQAEITQRL 120

OSY33027.1 LTQASRNANDGISIAQTTEGALNEINNNLQRVRELAVQSANSTNSQSDLDSIQAEITQRL 120

KWV07712.1 LTQASRNANDGISIAQTTEGALNEINNNLQRVRELAVQSANSTNSQSDLDSIQAEITQRL 120

AHA06007.1 LTQASRNANDGISIAQTTEGALNEINNNLQRVRELAVQSANSTNSQSDLDSIQAEITQRL 120

AAT81640.1 LTQASRNANDGISIAQTTEGALNEINNNLQRVRELAVQSANSTNSQSDLDSIQAEITQRL 120

***********************************:**::*.***:***.*** ** ***

ERH38463.1 EEIDRVSNQTQFNGVKVLSQDNQMKIQVGANDGETITIDLQKIDVKSLGLDGFNVNGPKE 180

AAT81621.1 EEIDRVSNQTQFNGVKVLSQDNQMKIQVGANDGETITIDLQKIDVKSLGLDGFNVNGPKE 180

OLP51747.1 NEIDRVSGQTQFNGVKVLAQDNTLTIQVGANDGETIDIDLKQINSQTLGLDSLNVQKAYD 180

APH26739.1 NEIDRVSGQTQFNGVKVLAQDNTLTIQVGANDGETIDIDLKQINSQTLGLDTLNVQKAYD 180

KNU33033.1 NEIDRVSGQTQFNGVKVLAQDNTLTIQVGANDGETIDIDLKQINSQTLGLDTLNVQKAYD 180

RAT91061.1 NEIDRVSGQTQFNGVKVLAQDNTLTIQVGANDGETIDIDLKQINSQTLGLDTLNVQKKYD 180

OSY33027.1 NEIDRVSGQTQFNGVKVLAQDNTLTIQVGANDGETIDIDLKQINSQTLGLDTLNVQQKYK 180

KWV07712.1 NEIDRVSGQTQFNGVKVLAQDNTLTIQVGANDGETIDIYLKQINSQTLGLDTLNVQQKYK 180

AHA06007.1 NEIDRVSGQTQFNGVKVLAQDNTLTIQVGANDGETIDIDLKQINSQTLGLDTLNVQQKYK 180

AAT81640.1 NEIDRVSGQTQFNGVKVLAQDNTLTIQVGANDGETIDIDLKQINSQTLGLDTLNVQQKYK 180

:******.**********:*** :.*********** * *::*: ::**** :**: .

ERH38463.1 ATVGDLKSS--F-KNVTGYDTYAAGADKYRVDINSGAVVTDAAAPDKVYVNAANGQL--- 234

AAT81621.1 ATVGDLKSS--F-KNVTGYDTYAAGADKYRVDINSGAVVTDDAAPDKVYVNAANGQL--- 234

OLP51747.1 VKDTAVTTK-AYADNGTTLDASGLDDAAIKAA-IGGTTGTAAVTGGTVKFDADNNKYFVT 238

APH26739.1 VSATAAMDPKSFTDGTKNL--TAPDATAIKAA-LGNPAATGDSLSATL--SFKDGKYYAT 235

KNU33033.1 VSATAAMDPKSFTNGTKNL--TAPDATAIKAA-LGNPATTGDSLSATL--SFKDGKYYAT 235

RAT91061.1 VDSTGVTQS-LDL-KTAGIT-----GATLKAGITGTTTETGSVKDGKVYYDADSKNYYVE 233

OSY33027.1 VSDTAATVT-GYTDSATAID-----KSTFAASATTL-GGTP-AITGDLKFDDTTGKYYAD 232

KWV07712.1 VSDTAATVT-GYADTTIALD-----NSTFKASATGL-GGTDQKIDGDLKFDDTTGKYYAK 233

AHA06007.1 VSDTAATVT-GYADTTIALD-----NSTFKASATGL-GGTDQKIDGDLKFDDTTGKYYAK 233

AAT81640.1 VSDTAATVT-GYADTTIALD-----NSTFKASATGL-GGTDQKIDGDLKFDDTTGKYYAK 233

. . * : . :

ERH38463.1 -----TTDDAENNTAVDLFKTTKSTAGTAEAKAIAGAIKGGKEGDTFDYKGVTFTIDTKT 289

AAT81621.1 -----TTDDAENNTAVNLFKTTKSTAGTDEAKAIASAIKGGKEGDTFDYKGVSFTIDTKA 289

OLP51747.1 IGGFTG-ADAAKNG---DYEVNVATDG--KVTLATGATKTTMPAG-----AATK-TEVQE 286

APH26739.1 VAGYTNAADTSKNG---KYEVNVDSAT-GAVTFNAAPTKATVT-G-----DTTV---TKV 282

KNU33033.1 VAGYTNAADTSKNG---KYEVNVDSAT-GAVTFNAAPTKATVT-G-----DTTV---TKV 282

RAT91061.1 VDFTDTTDKAAHAG---FYKADVD-AD-GNVSLATGATKEAK--------PTNAVEVEKT 280

OSY33027.1 V--SGTTA---KDG---VYEVTVA-AD-GKVTLTGTPTGPITAGF-----PSTATKDVKQ 277

KWV07712.1 VTVTGETG---KNG---YYEVSVNKTN-GEVTLAGGATSPLTGGL-----PATATEDVKN 281

AHA06007.1 VTVTGGTG---KDG---YYEVSVDKTN-GKVTLAGGATSPLTGGL-----PATATEDVKN 281

AAT81640.1 VTVTGGTG---KDG---YYEVSVDKTN-GKVTLAGGTTSPLTGGL-----PATATEDVKN 281

: ::. .. . :

ERH38463.1 GDDGNGKVSTTINGEKVTLTVADIATGATDVNAATLQSSKNVYT----SVVNGQFTFDDK 345

AAT81621.1 GDDGNGTVSTTINGEKVTLTVADITAGAANVNDATLQSSKNVYT----SVVNGQFTFDDK 345

OLP51747.1 LKDTPAVVSADA---KNAL----IAGGVDTADANGAELVKMSYTDKNGKTIEGGYALKAG 339

APH26739.1 QVNAPVAVSTDV---KKAL----EDGGVSNADATAAKLVKMSYTDKNGKSIDGGYALEAG 335

KNU33033.1 QVNAPVAVSTDV---KKAL----EDGGVSNADATAAKLVKMSYTDKNGKSIDGGYALEAG 335

RAT91061.1 IDEKPLKASSSV---QDAL----KASGIADAVAEAATVVKMSYTDKNGKTIDGGYGIKVG 333

OSY33027.1 TQQEN-ADLTEA---KAAL----TAAGV--AAAGTASVVKMSYTDNNGKTIDGGLAVKVG 327

KWV07712.1 VQVAN-ADLTEA---KAAL----TAAGV----TGTASVVKMSYTDNNGKTIDGGLAVKVG 329

AHA06007.1 VQVAN-ADLTEA---KAAL----TAAGV----TGTASVVKMSYTDNNGKTIDGGLAVKVG 329

AAT81640.1 VQVAN-ADLTEA---KAAL----TAAGV----TGTASVVKMSYTDNNGKTIDGGLAVKVG 329

: : :* * * ** . ::* ..

ERH38463.1 TKNESAKLSDLEANNAVKGESKITVNGAEYTAN---------------ATGDKITLAGKT 390

AAT81621.1 TKNESAKLSDLEANNAVKGESKITVNGAEYTAN---------------AAGDKVTLAGKT 390

OLP51747.1 DKYYAA--------DYDEATGAIKAKTTSYIAADGTTKTAANQLGGVDGKTEVVTIDGKT 391

APH26739.1 GKYYAA--------TYDEGTGKITANVTTYTDSTGVTKTAANQLGGVDGKTEVVTIDGKT 387

KNU33033.1 GKYYAA--------TYDEGTGKITANVTTYTDSTGATKTAANQLGGVDGKTEVVTIDGKT 387

RAT91061.1 DDYYAA--------TKE-KDGSYSINSTSYTDKDGNTKTALNQLGGADGKTEVVSIDGKT 384

OSY33027.1 DDYYSA--------TQN-KDGSISINTTKYTADDGTSKTALNKLGGADGKTEVVSIGGKT 378

KWV07712.1 DDYYSA--------TQN-KDGSISINTTKYTADDGTSKTALNKLGGADGKTEVVSIGGKT 380

AHA06007.1 DDYYSA--------TQN-KDGSISINTTKYTADDGTSKTALNKLGGADGKTEVVSIGGKT 380

AAT81640.1 DDYYSA--------TQN-KDGSISINTTKYTADDGTSKTALNKLGGADGKTEVVSIGGKT 380

. :* . . : : * . : ::: ***

ERH38463.1 MFIDKTASGVSTLINEDAAAAKKSTANPLASIDSALSKVDAVRSSLGAIQNRFDSAITNL 450

AAT81621.1 MFIDKTASGVSTLINEDAAAAKKSTANPLASIDSALSKVDAVRSSLGAIQNRFDSDITNL 450

OLP51747.1 YNASKAAGHDFKAQPELAEAAAKTTENPLQKIDAALAQVDALRSDLGAVQNRFNSAITNL 451

APH26739.1 YNASKAAGHDFKAQPELAEAAAKTTENPLAKIDAALAQVDALRSDLGAVQNRFNSAITNL 447

KNU33033.1 YNASKAAGHDFKAQPELAEAAAKTTENPLAKIDAALAQVDALRSDLGAVQNRFNSAITNL 447

RAT91061.1 YNASKAAGHNFKAQPDLAEAAATTTENPLQKIDAALAQVDALRSDLGAVQNRFNSAITNL 444

OSY33027.1 YAASKAEGHNFKAQPDLAEAAATTTENPLQKIDAALAQVDTLRSDLGAVQNRFNSAITNL 438

KWV07712.1 YAASKAEGHNFKAQPDLAEAAATTTENPLQKIDAALAQVDTLRSDLGAVQNRFNSAITNL 440

AHA06007.1 YAASKAEGHNFKAQPDLAEAAATTTENPLQKIDAALAQVDTLRSDLGAVQNRFNSAITNL 440

AAT81640.1 YAASKAEGHNFKAQPDLAEAAATTTENPLQKIDAALAQVDTLRSDLGAVQNRFNSAITNL 440

.*: . . : * ** .:* *** .**:**::**::**.***:****:* ****

ERH38463.1 GNTVTNLNSARSRIEDADYATEVSNMSKAQILQQAGTSVLAQANQVPQNVLSLLR 505

AAT81621.1 GNTVTNLNSARSRIEDADYATEVSNMSKAQILQQAGTSVLAQANQVPQNVLSLLR 505

OLP51747.1 GNTVNNLSEARSRIEDSDYATEVSNMSRAQILQQAGTSVLAQANQVPQNVLSLLR 506

APH26739.1 GNTVNNLSEARSRIEDSDYATEVSNMSRAQILQQAGTSVLAQANQVPQNVLSLLR 502

KNU33033.1 GNTVNNLSEARSRIEDSDYATEVSNMSRAQILQQAGTSVLAQANQVPQNVLSLLR 502

RAT91061.1 GNTVNNLSSARSRIEDSDYATEVSNMSRAQILQQAGTSVLAQANQVPQNVLSLLR 499

OSY33027.1 GNTVNNLTSARSRIEDSDYATEVSNMSRAQILQQAGTSVLAQANQVPQNVLSLLR 493

KWV07712.1 GNTVNNLTSARSRIEDSDYATEVSNMARAQILQQAGTSVLAQANQVPQNVLSLLR 495

AHA06007.1 GNTVNNLTSARSRIEDSDYATEVSNMSRAQILQQAGTSVLAQANQVPQNVLSLLR 495

AAT81640.1 GNTVNNLTSARSRIEDSDYATEVSNMSRAQILQQAGTSVLAQANQVPQNVLSLLR 495

****.**..*******:*********::***************************

**B**

**
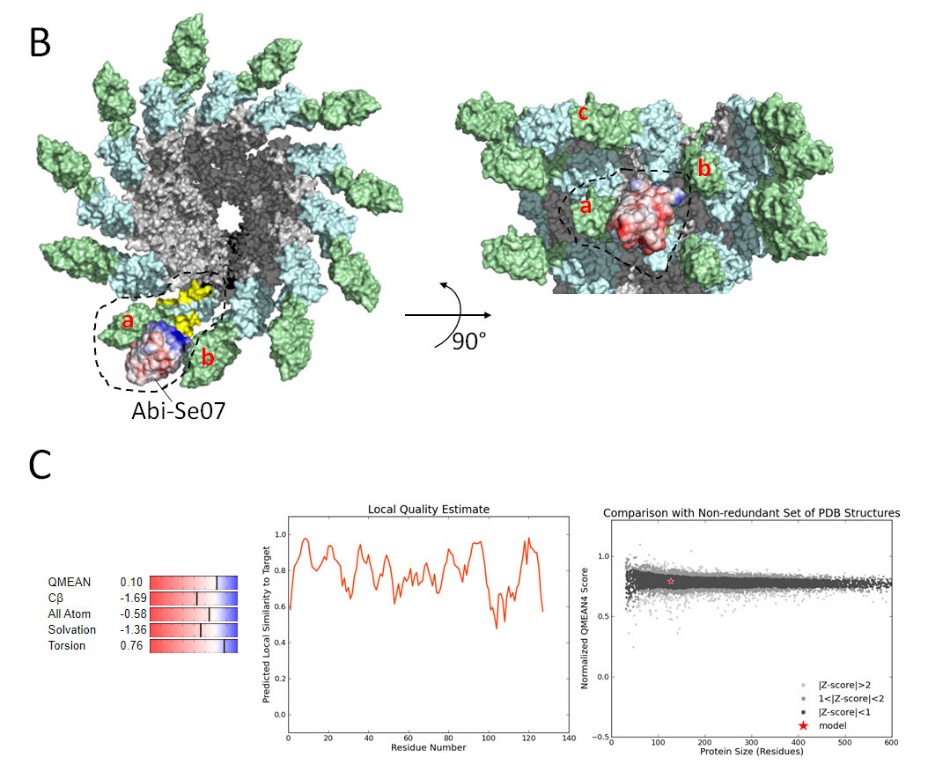
**

**Supplementary Figure S1. A.** Alignment of *S. enterica* serovar Hadar (RAT91061.1), Typhimurium (AHA06007.1), Heidelberg (AAT81640.1), Enteritidis (ERH38463.1), Newport (APH26739.1), Javiana (OLP51747.1), Senftenberg (ABR18492.1). Kentucky (KWV07712.1), Infantis (OSY33027.1), and Saint-Paul (KNU33033.1). Domains D0, D1, D2, and D3 are colored black, grey, cyan, and green, respectively. Underlined region corresponds to residues within 4 Å of Abi-Se07 CDRs in the simulated docking solution. **B.** Visualization of docking of Abi-Se07 to FliC. This docking is inaccurate due to low quality modeling of CDRs (see C) and is provided merely as a to-scale visualization. **C.** SWISS-MODEL QMEAN quality score relative to all non-redundant structures in the protein data bank (left and right panels). Local quality estimate along the length of the sdAb polypeptide (middle panel). Quality estimate declines around residue 100 to 120, corresponding to CDR3.


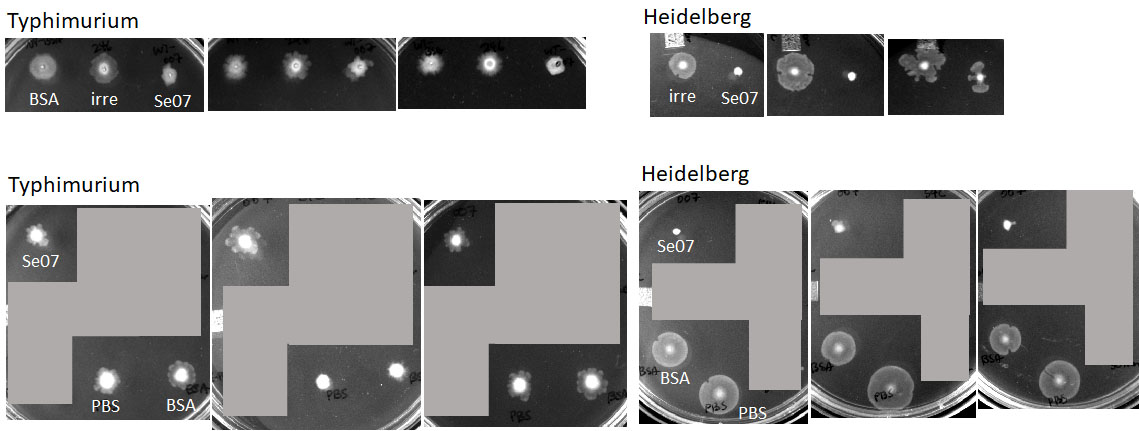


**Supplementary Figure S2.** Motility of *S. enterica* was assessed for serovars Typhimurium and Heidelberg. Three replicates were conducted for each set of experiments. In the presence of BSA, PBS or irrelevant sdAb, Typhimurium exhibited inconsistent motilities while Heidelberg exhibited consistent and reproducible motilities with the control treatment.
